# Sex Disparities in ICH: Presentation, Care, and Outcomes in the TICH-2 Trial

**DOI:** 10.1159/ced/accag015

**Published:** 2026-10-01

**Authors:** Chaamanti S. Menon, Michael Desborough, Elinor Mai Greenman, Lisa Woodhouse, Zhe Kang Law, Kailash Krishnan, Rob A. Dineen, Philip M. Bath, Nikola Sprigg, on behalf of TICH 2 investigators

**Affiliations:** a Stroke Trials Unit, University of Nottingham, Nottingham, UK; b Stroke, Department of Stroke Medicine, Queen’s Medical Centre, Nottingham Hospital University Trust, Nottingham, UK; c Department of Clinical Haematology, Oxford University Hospitals NHS Foundation Trust, Oxford, UK; d Radcliffe Department of Medicine, University of Oxford, NHS Blood and Transplant, John Radcliffe Hospital, Oxford, UK; e School of Medicine, University of Nottingham, Nottingham, UK; f Neurology Unit, Department of Medicine, UKM Medical Centre, Hospital Canselor Tuanku Muhriz, National University of Malaysia, Nottingham, UK; g Radiological Sciences, Mental Health and Clinical Neuroscience, University of Nottingham, Nottingham, UK; h NIHR Nottingham Biomedical Research Centre, Nottingham, UK

**Keywords:** Intracerebral haemorrhage, Sex disparities, Stroke outcomes, Tranexamic acid, TICH-2 trial, Modified Rankin Scale, Processes of care, Treatment escalation plan

## Abstract

**Introduction:**

Sex-based disparities in ischaemic stroke outcomes are more widely reported than those for intracerebral haemorrhage. This study aims to examine sex differences in baseline characteristics, imaging findings, care processes, and functional outcomes following ICH and to determine whether the treatment effect of tranexamic acid (TXA) differed by sex.

**Methods:**

We performed a post hoc analysis of the TICH-2 trial, a multicentre, double-blind, randomised, placebo-controlled study of TXA in spontaneous ICH. Baseline characteristics and care processes were compared between men and women. The primary outcome was functional status at day 90 (modified Rankin Scale, mRS). Regression models were adjusted for prespecified prognostic variables. Odds ratios represent the odds of poor outcome in women compared to men, with men serving as the reference group. Interaction terms were used to test for sex-treatment effects.

**Results:**

Of 2,325 patients in TICH-2, 1,024 (44%) were women. Women were older (mean age 72.3 vs. 66.2 years, *p* < 0.001), had worse pre-stroke function (mRS 0.71 vs. 0.43, *p* < 0.001), larger haematomas (25.6 mL vs 22.7 mL, *p* = 0.012), and more lobar bleeds (39.2% vs. 25.9%, *p* < 0.001). They were also more likely to receive early do-not-attempt-resuscitation (DNAR) orders (22.6% vs. 12.4%, *p* = 0.003). After adjustment, there were no significant sex differences in functional outcome at 90 days (aOR 1.04, 95% CI 0.88–1.22). There was no evidence of a sex-specific treatment effect with TXA (interaction *p* > 0.1).

**Conclusion:**

Despite older age, poorer pre-stroke function, more severe imaging features, and higher rate of early DNAR status, women had similar adjusted 90-day functional outcomes to men.

## Introduction

Sex disparities in stroke outcomes and care processes, particularly in intracerebral haemorrhage (ICH), are poorly understood. In studies looking at stroke overall including ischaemic stroke and ICH, men typically have a higher incidence of stroke across most age groups [1–3]. However women tend to have poorer outcomes; although they are more likely to survive, they are also more likely to live with disability and report lower quality of life, particularly after the age of 75 years [4]. The UK Biobank cohort shows that hypertension, smoking, and lower socioeconomic status is associated with worse stroke outcomes in women [5]. In ischaemic stroke, some data suggest that women may be less likely to receive thrombolysis and other acute treatments, though this is inconsistently reported [4, 6–9].

In contrast, sex differences in ICH are less well characterised. Recent evidence includes a systematic review and meta-analysis of 32 observational studies reporting similar mortality but poorer functional outcomes in women after ICH [10]. However, most included studies were retrospective, heterogeneous in design, and lacked consistent adjustment for key prognostic factors such as haematoma characteristics and acute care processes. As pooled analyses were based on study-level rather than patient-level data, it remains unclear whether observed differences reflect biological sex, baseline health status, haemorrhage severity, or variation in care [10]. Some were also conducted before recent advances in ICH care, reducing their relevance to current clinical practice [11, 12]. Consequently, the extent to which sex differences in baseline characteristics or care processes influence outcomes after ICH remains unclear.

To address this gap, we performed a post hoc analysis of the Tranexamic Acid for Intracerebral Haemorrhage 2 (TICH-2) trial [13]. This large, international randomised controlled trial used standardised imaging, protocol-driven management, and patient-level data, providing a unique opportunity to evaluate sex-based differences in a controlled setting. Specifically, we examined whether outcomes after ICH differ by sex and whether disparities exist in baseline characteristics, radiological features, care processes such as do-not-attempt-resuscitation (DNAR) decisions, intensive care unit admissions, and response to tranexamic acid (TXA) treatment.

## Methods

We used data from the large TICH-2 trial, an international, double-blind, placebo-controlled study of TXA in spontaneous ICH [13, 14]. The full trial design and procedures have been published previously [15, 16]. In brief, adults presenting within 8 h of stroke symptom onset were randomised in a 1:1 ratio to receive either TXA 1 g as a bolus followed by 1 g as an infusion over 8 h, or matching placebo, stratified by country and minimised for key prognostic variables. Exclusion criteria included secondary causes of haemorrhage, contraindications to TXA, coma, or end-of-life care. Participants in this trial were enrolled based on their capacity and the urgency of the situation. If full written consent was not possible, a tiered consent process was followed using brief consent from the patient, a legal representative, or an independent doctor, with full informed consent obtained later [17].

Baseline data included demographics, medical history, pre-stroke modified Rankin Scale, stroke severity (National Institutes of Health stroke scale, NIHSS), consciousness level (Glasgow coma scale, GCS), and imaging features such as haematoma volume, location, intraventricular extension, and other brain imaging features as listed in Table 2 [18]. Follow-up assessments were conducted at days 2 and 7 for collection of research and clinical care processes such as blood pressure control, neurocritical care involvement, early DNAR placement, and early neurological deterioration (defined as NIHSS increase of ≥ 4 or a decline in GCS of ≥ 2 by day 7) [19]. Centralised, blinded assessor telephone follow-up was performed at 90 days for all participants and at 365 days for patients from the UK only. International follow-up was not possible due to logistical and funding restrictions at 365 days [20]. At days 90 and 365, evaluation of clinical severity, functional outcomes, and health-related quality of life, cognition as well mood using the modified Rankin Scale, EQ-5D, Barthel index, telephone interview cognition state (TICS-M), and Zung depression scale (ZDS) was undertaken. Serious adverse events were recorded prospectively and adjudicated centrally. If functional outcome (mRS) was not received at the time of follow-up, their GP was contacted to help assess current functional outcome.

### Statistical Analyses

Statistical analyses were performed in SPSS version 22. Differences between men and women were assessed using chi-squared tests for categorical variables and *t* tests or Mann-Whitney U tests for continuous variables. Ordinal outcomes such as the modified Rankin Scale were analysed using ordinal logistic regression and binary outcomes such as mortality, haematoma expansion, ICU admission, and DNAR status using binary logistic regression. Time-to-event data were analysed using Cox proportional hazards models, and continuous outcomes using general linear models.

All models were adjusted for the prespecified covariates in the TICH-2 minimisation algorithm: age, pre-stroke modified Rankin Scale, baseline systolic blood pressure, NIHSS score, baseline ICH volume, time from onset to randomisation, intraventricular haemorrhage, antiplatelet use, and treatment allocation. To examine treatment-by-sex interactions, we included an interaction term between sex and treatment group (TXA versus placebo) in regression models for haematoma expansion, early death, and functional outcomes. Results are presented as odds ratios, hazard ratios, or mean differences with 95% confidence intervals. Odds ratios represent the odds of poor outcome in women compared to men, with men serving as the reference group. Missing data were not imputed.

## Results

A total of 2,325 participants were enrolled in the trial and 2,307 were included in the primary analysis of this paper (Fig. 1). At 90 days, 24 mRS assessments were missing for male and 14 for female. After GP mRS assessments were received, overall mRS missing in male was 10 (0.8%) and 8 (1%) in females (Fig. 1). The trial was composed of 1,301 men (56%) and 1,024 women (44%). Women were significantly older than men at baseline (mean age 72.3 vs. 66.2 years, *p* < 0.001) and had worse pre-stroke functional status (mRS 0.71 vs. 0.43, *p* < 0.001), higher severity (NIHSS 13 vs. 12, *p* = 0.002), and lower conscious level (GCS 14 vs. 15, *p* = 0.004) (Table 1). Men were more likely to weigh more (83.9 kg vs. 66.8 kg, *p* < 0.001) and a higher percentage of men had vascular risk factors such as ischemic heart disease (10.8% vs. 6.1%, *p* < 0.001), diabetes mellitus (16.7% vs. 9.3%, *p* < 0.001), and smoking (21.4% vs. 9.7%, *p* < 0.001) (Table 1). Onset to randomisation (*p* = 0.897) and onset to CT scan time (*p* = 0.266) were not significantly different. Radiologically, women had larger haematoma volumes at baseline (25.6 mL vs. 22.7 mL, *p* = 0.012) (Table 2) and day 2 (28.6 mL vs. 25.6 mL, *p* = 0.014) (Table 4) and more lobar haemorrhages (39.2% vs. 25.9%, *p* < 0.001) (Table 2). Markers of frailty in the brain such as leukoaraiosis were more common in women (Table 2). Men were more likely to have deeper bleeds (54.4% vs. 71%, *p* < 0.001) (Table 2).

**Fig. 1.**
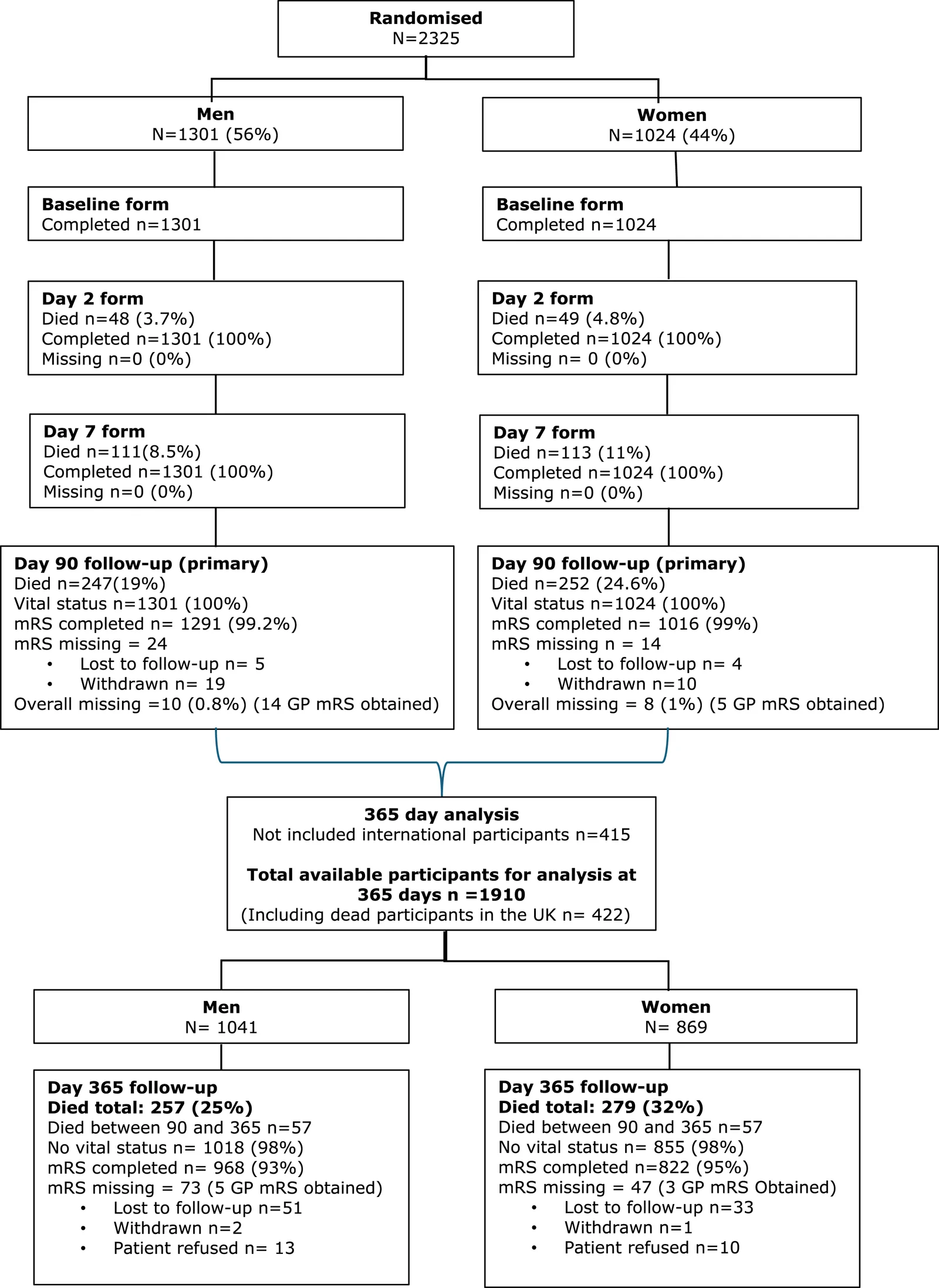
Flowchart showing the number of patients screened, randomised in the TICH-2 trial, and included in this post hoc sex disparities analysis.

**Table 1.** Differences in baseline demographics

Baseline clinical features	Women	Men	Chi square/*t* test (CI of the difference)	*p* value
	*N* = 1,024	*N* = 1,301		
Age, mean (SD), years	72 (13.5)	66 (13.5)	−10.7 (−7.2 to 4.9)	**0.001**
Ethnicity, *n* (%)	​	​	10.8	0.29
(1) White	897 (87.6)	1,081 (83.1)		
(2) Black	39 (3.8)	57 (4.4)		
(3) Asian	78 (7.6)	140 (10.8)		
(4) Other	10 (1)	22 (1.7)		
Systolic blood pressure, mean (SD), mm Hg	173.0 (26.9)	172.2 (27.4)	−2.7 (−5.2–−0.8)	**0.008**
Diastolic blood pressure, mean (SD), mm Hg	91.8 (17.7)	94.4 (18.4)	4.8 (2.1–5.1)	**0.001**
Pre-stroke mRS (out of 6)	​	​	−6.7	**0.001**
Mean (SD)	0.7 (1.1)	0.4 (0.9)		
Median (IQR)	0 (0–4)	0 (0–4)		
NIHSS at baseline	13 (7–19)	12 (6–18)	−3.7 (−1.8–0.5)	**0.001**
Median out of 42 (IQR)				
GCS at baseline	15 (12–15)	14 (13–15)	4.0 (0.2–0.5)	**0.004**
Median (IQR)				
Onset to randomisation	3.9 (1.7)	3.9 (1.7)	−0.1 (−1.5 to 0.1)	0.897
Mean (SD)				
Door to scan time	2.3 (1.3)	2.3 (1.3)	−0.5 (−0.13 to 0.08)	0.266
Mean (SD)				
TXA given, *n* (%)	505 (49.3)	519 (50.7)	0.4	0.552
Weight (mean, SD) [total *n*], Kg	66.8 (16.7) [*n* = 732]	83.9 (17.8) [*n* = 973]	20.3 (15.5–18.5)	**0.001**
Previous ischaemic stroke or TIA, *n* (%)	131 (12.7)	198 (15.2)	5.2	0.076
Previous antiplatelet therapy, *n* (%)	245 (23.9)	366 (28.1)	6.1	0.048
Previous statin use, *n* (%)	254 (24.8)	367 (28.2)	3.8	0.15
History of ischaemic heart disease, *n* (%)	62 (6.5)	140 (10.8)	0.6	**0.001**
History of hypertension, *n* (%)	626 (61.3)	794 (61.3)	1.9	0.990
History of diabetes mellitus, *n* (%)	95 (9.3)	217 (16.7)	28.7	**0.001**
History of smoking, *n* (%)	​	​	129.9	**<0.001**
(1) Previous smoker	1) 232 (22.7)	1) 442 (34.0)		
(2) Current smoker	2) 99 (9.7)	2) 278 (21.4)		
(3) Never smoked	3) 625 (61.0)	3) 526 (40.4)		
History of hyperlipidaemia, *n* (%)	248 (24.2)	346 (26.6)	1.9	0.192
Current alcohol intake, mean (SD) [total *n*], units	6.21 (16.3) [*n* = 895]	11.83 (26.6) [*n* = 1,154]	8.8 (6.9–10.8)	**<0.001**
Underlying cause, *n* (%)	​	​	24.0	**0.002**
(1) Amyloid angiopathy	1) 52 (5.1)	1) 28 (2.2)		
(2) AVM	2) 13 (1.3)	2) 12 (0.9)		
(3) Tumour	3) 3 (0.3)	3) 2 (0.2)		
(4) Aneurysm	4) 10 (1.0)	4) 3 (0.2)		
(5) Venous infarct	5) 1 (0.1)	5) 4 (0.3)		
(6) Coagulopathy	6) 3 (0.3)	6) 4 (0.3)		
(7) Hypertension	7) 472 (46.1)	7) 649 (49.9)		
(8) Other/not recorded	8) 470 (45.9)	8) 599 (46.0)		

**Table 2.** Differences in radiological baseline factors

	Women	Men	Chi-squared/*T* test	*p* value
	*N* = 1,024	*N* = 1,301		
Haematoma location (*n*)	1,010	1,284	71.7	**<0.001**
Supratentorial deep, *n* (%)	550 (54.5)	912 (71.0)		
Supratentorial lobar, *n* (%)	397 (38.8)	296 (23.1)		
Infratentorial, *n* (%)	68 (6.7)	76 (5.9)		
CT angiography (*n*)	100	149	3.6	0.17
Spot positive, *n* (%)	18 (1.8)	37 (2.8)		
Spot negative, *n* (%)	82 (8.0)	111 (8.5)		
CT non-contrast signs (*n*)	784	1,006		
Blend sign, *n* (%)	143 (15.2)	180 (15.4)	0.02	0.89
Black hole sign, *n* (%)	150 (19.2)	148 (14.5)	7.2	0.07
Island sign, *n* (%)	61 (13.0)	62 (10.0)	2.4	0.12
Intracerebral haematoma volume at baseline (*n*)	1,001	1,272	−2.8 (−5.5 to −1.01)	0.05
Mean (SD), mL	25.8 (28.1)	22.5 (26.3)		
Intraventricular haemorrhage (*n*)	1,014	1,288	0.6	0.45
IVH present, *n* (%)	305 (30.1)	369 (28.6)		
IVH present, *n* (%)				
Intraventricular haemorrhage volume, mean (SD), mL	9.8 (9.02)	8.28 (10.7)	−0.5	0.64
Perihaematomal oedema, mean (SD), mL	15.0 (17.8)	12.6 (15.1)	−3.3	**<0.001**
Midline shift, mean (SD), mL	5.94 (2.3)	5.90 (2.9)	−0.1	0.89
Hydrocephalus (*n*)	351	442	51.7	**<0.001**
Not present, *n* (%)	102 (29.1)	139 (31.4)		
Mild, *n* (%)	183 (52.1)	235 (53.2)		
Moderate, *n* (%)	46 (13.1)	53 (12)		
Severe, *n* (%)	20 (5.7)	15 (3.4)		
Subarachnoid haemorrhage (*n*)	1,014	1,288	29.8	**<0.001**
*n* (%)	185 (18.2)	133 (10.3)		
Brain tissue reduction (*n*)	1,019	1,292		
Cortical atrophy, *n* (%)				
Mild	784 (84.4)	1,024 (87.1)	2.8	0.25
Severe	142 (15.3)	146 (12.4)	0.04	0.85
Central atrophy, *n* (%)				
Mild	200 (21.5)	289 (24.6)		
Severe	54 (5.8)	63 (5.4)		
Periventricular lucencies, *n* (%)	1,019	1,292	10.4	**0.001**
	512 (50.2)	562 (43.5)		
Old cortical lesions, *n* (%)	929	1,175	2.2	0.14
	513 (87.4)	675 (84.6)		

Completion rates for baseline, day 2, and day 7 forms were 100% for both sexes (Fig. 1). At day 90, follow-up was achieved in 100% of participants for vital status, with modified Rankin Scale (mRS) data available in 99.2% of men and 99% of women (Fig. 1). By day 365, after excluding international participants, 1,910 patients were eligible for analysis (1,041 men and 869 women). Follow-up completeness at 1 year remained high, with mRS data available for 93% of men and 95% of women (Fig. 1). There was no meaningful difference in follow-up completeness between men and women at any time point.

In clinical care processes, blood pressure management by day 2 did not differ between men and women. Mean systolic blood pressure, change from baseline, use of antihypertensive therapy, and timing of treatment initiation were similar (all *p* > 0.05) (Table 3). Routes of blood pressure medication administration were also comparable, except for oral therapy, which was more frequent in men (45.2% vs. 35.5%, *p* < 0.001) (Table 3). Men were more likely to receive neurosurgery (5.7% vs. 4.6%), intensive care unit admission (11.3% vs. 8.3%), and ventilation (8.2% vs. 5.8%), but these differences were not significant after adjustment (Table 3). DNAR orders were significantly more frequent in women within the first 48 h (22.6% vs. 12.4%, OR 1.54, 95% CI 1.16–2.05, *p* = 0.003), and this remained true at day 7 even after adjustment in women (28.5% vs. 16.7%, OR 1.41, *p* = 0.01) (Table 3).

**Table 3.** Differences in processes of care acute treatment and discharge outcomes

​	Women	Men	Adjusted MD/chi square (χ^2^)/adjusted OR (95% CI)	*p* value
	*N* = 1,024	*N* = 1,301		
BP control				
Systolic BP day 2, mean (SD), mm Hg	150 (21.3)	148.8 (19.3)	MD 0.3 (−1.31 to 1.91)	0.715
Change from mean baseline systolic blood pressure, mean (SD), mm Hg	−23.5 (27.6)	−20.5 (25.9)	MD 0.83 (−1.26 to 2.92)	0.436
Any BP lowering therapy by day 2, *n* (%)	740 (72.7)	996 (76.7)	χ^2^ 4.8	0.28
Onset until first medication received from randomisation, mean (SD), h	4.36 (1.83)	4.38 (1.76)	MD 0.01 (−0.02 to 0.04)	0.38
Onset from first medication dose to second dose, mean (SD), h	0.30 (0.35)	0.27 (0.30)	MD 0.03 (−0.003 to 0.54)	0.07
IV route, *n* (%)	577 (56.3)	722 (55.5)	χ^2^ 0.17	0.681
NG route, *n* (%)	38 (3.7)	52 (4)	χ^2^ 0.13	0.723
Patch route, *n* (%)	105 (10.3)	130 (10)	χ^2^ 0.04	0.835
Oral route, *n* (%)	364 (35.5)	588 (45.2)	χ^2^ 22.1	**<0.001**
Acute treatment				
Neurosurgery at 7 days, *n* (%)	47 (4.6)	74 (5.7)	Binary OR 1.03 (0.69–1.56)	0.87
Intensive care (ITU) at 7 days, *n* (%)	85 (8.3)	147 (11.3)	Binary OR 0.93 (0.69–1.27)	0.65
Ventilation at 7 days, *n* (%)	59 (5.8)	107 (8.2)	Binary OR 0.87 (0.61–1.25)	0.45
Do not Resuscitate order (DNAR) at day 2, *n* (%)	231 (22.6)	161 (12.4)	Binary OR 1.54 (1.16–2.05)	**0.003**
DNAR at day 7, *n* (%)	292 (28.5)	217 (16.7)	Binary OR 1.41 (1.10–1.84)	**0.01**

The primary outcome, mRS, at 90 days following adjustment was aOR 1.04 (95% CI 0.88–1.22, *p* = 0.602). Sensitivity analyses using a binary measure, mRS >3, also showed no significant difference (OR 1.14, 95% CI 0.86–1.46, *p* = 0.318) (Table 4). When unadjusted, the distribution in the mRS shows that women had worse outcomes at day 90 and 1 year (Fig. 2). Mortality at day 2 and day 7 was not significantly different between the sexes (Table 4). Women had higher rates of death by 90 days (24.6% vs. 19.0%); the differences were not statistically significant after adjustment (HR at 90 days 0.89, 95% CI 0.73–1.08, *p* = 0.23 [Table 4; Fig. 3a]).

**Table 4.** Primary and secondary outcomes

	Women	Men	Adjusted OR/MD/HR (95% CI)	*p* value
Primary outcome, mRS at day 90				
Total number of participants with outcome (mRS/6), *n* (%)	**1,016**	**1,291**		
mRS 0	17 (1.7)	33 (2.6)	Ordinal OR 1.04 (0.88–1.22)	0.602
mRS 1	89 (8.8)	150 (11.6)		
mRS 2	130 (12.8)	248 (19.2)		
mRS 3	156 (15.4)	225 (17.4)		
mRS 4	209 (20.6)	225 (17.4)		
mRS 5	163 (16.0)	163 (12.6)		
mRS 6, death	252 (24.8)	247 (19.1)		
Sensitivity analysis				
mRS, median [IQR]	4 [3]	3 [4]		
mRS, mean (SD)	3.9 (1.7)	3.5 (1.7)		
mRS >3, *n* (%)	780 (76.8)	860 (66.6)	Binary OR 1.14 (0.86–1.46)	0.318
Secondary outcomes				
Early deterioration (defined as a rise in NIHSS by 4 points and/or a fall in GCS by 2 points)	**947**	**1,198**		
Neurological deterioration by day 2, *n* (%)	212 (22.4)	214 (17.9)	Binary OR 1.10 (0.85–1.41)	0.477
Neurological deterioration by day 7, *n* (%)	159 (19.2)	189 (17.3)	Binary OR 0.98 (0.74–1.28)	0.869
Radiological outcomes	**937**	**1,204**		
CT scans at day 2	929 (91.1)	1,210 (93.1)	X^2^ 3.42	0.06
Intracerebral haematoma volume at day 2, mean (SD), mL	28.577 (32.456)	25.617 (29.608)	*T* test −2.46 (−5.95 to −0.67)	0.014
Intracerebral haematoma absolute growth, mean (SD), mL	4.39 (16.4)	4.25 (15.5)	MD 0.14 (−1.51 to 1.23)	0.52
Intracerebral haematoma expansion (>33% or >6 mL), *n* (%)	235 (25.3)	334 (28.2)	Binary OR 0.77 (0.62–0.95)	**0.02**
Mortality outcomes	**1,024**	**1,301**		
Day 2				
Death by day 2, *n* (%)	49 (4.8)	48 (3.7)	Binary OR 1.07 (0.68–1.69)	0.79
Day 7				
Death by day 7, *n* (%)	113 (11)	111 (8.5)	Binary OR 0.99 (0.70–1.38)	0.93
Day 90				
Death by day 90, *n* (%)	252 (24.6)	247 (19)	Binary OR 0.86 (0.66–1.12)	0.26
Survival analysis by day 90, *n* (%)	252 (24.6)	247 (19)	HR 0.86 (0.72–1.04)	0.23
Serious adverse outcomes* at day 90	**1,024**	**1,301**		
All SAEs	504 (49.2)	573 (44)	Binary OR 0.92 (0.76–1.12)	0.44
Discharge	**827**	**1,103**		
Length of stay, mean (SD), days	23.8 (27.9)	20.9 (26.2)	MD 1.00 (−3.39 –to 1.38)	0.41
Disposition at discharge, *n* (%)				
Home	374 (36.6)	544 (41.8)	Binary OR 1.12 (0.91–1.37)	0.30
Institution	452 (44.2)	559 (43)	Binary OR 1.00 (0.84–1.19)	1.00
Hospital	197 (19.3)	198 (15.2)	Binary OR 0.88 (0.66–1.16)	0.36
Disposition at 90 days, *n* (%)				
Home	514 (67.8)	757 (73.5)	Binary OR 1.01 (0.78–1.29)	0.96
Institution	91 (12.0)	48 (4.7)	Binary OR 1.67 (1.11–2.51)	**0.01**
Hospital	153 (20.2)	225 (21.8)	Binary OR 0.79 (0.61–1.03)	0.09
Mental health and quality of life outcomes at day 90				
EQ-5D HUS (/1), mean (SD)	0.29 (0.38)	0.38 (0.40)	MD −0.01 (−0.02 to 0.04)	0.44
EQ-VAS (/100), mean (SD)	44.7 (33.7)	51.7 (33.0)	MD −0.49 (1.85–2.83)	0.015
Barthel Index (/100), mean (SD)	47.1 (43.4)	57.8 (43.7)	MD −0.30 (2.93–2.32)	0.70
TICS-M (/39), mean (SD)	11.8 (12.9)	15.8 (12.3)	MD −0.32 (−1.3 to 0.63)	0.51
ZDS (/100), mean (SD)	72.1 (29.6)	63.7 (29.2)	MD 0.72 (−3.04 to 1.61)	0.55
Functional outcome at day 365				
Total number of participants with outcome (mRS/6), *n* (%)	**822**	**968**		
mRS 0	20 (2.4)	24 (2.5)	Ordinal OR 1.05 (0.87–1.23)	0.63
mRS 1	74 (9.0)	153 (15.8)		
mRS 2	91 (11.1)	161 (16.6)		
mRS 3	135 (16.4)	179 (18.5)		
mRS 4	139 (16.9)	115 (11.9)		
mRS 5	84 (10.2)	79 (8.2)		
mRS 6, death	279 (33.9)	257 (26.5)		
Sensitivity analysis				
mRS, median [IQR]	4 (3)	3 (4)		
mRS, mean (SD)	4 (1.8)	3.5 (1.9)		
mRS >3, *n* (%)	502 (61.1)	451 (46.6)	Binary OR 0.80 (0.62–1.03)	0.077
Mortality outcomes at day 365	**855**	**1,018**		
Death by day 365, *n* (%)	279 (32%)	257 (25%)	Binary OR 0.85 (0.65–1.12)	0.250
Survival analysis day 365, *n* (%)	279 (32%)	257 (25%)	HR 0.87 (0.72–1.04)	0.62
Disposition at 365 days, *n* (%)				
Home	428 (80.5)	623 (89.6)	Binary OR 0.70 (0.48–1.03)	0.08
Institution	93 (17.5)	68 (9.8)	Binary OR 1.28 (0.85–1.91)	0.23
Hospital	11 (2.1)	4 (0.6%)	Binary OR 2.90 (0.87–9.71)	0.08
Mental health and quality of life outcomes at day 365				
EQ-5D HUS (/1), mean (SD)	0.38 (0.4)	0.48 (0.4)	MD −0.03 (−0.01 to 0.07)	0.175
EQ-VAS (/100), mean (SD)	57.2 (29.03)	61 (27.7)	MD 0.36 (−3.39 to 2.68)	0.816
Barthel Index (/100), mean (SD)	70.1 (34.8)	79.5 (31.1)	MD −0.37 (−1.68 to 4.55)	0.367
TICS-M (/39), mean (SD)	18.17 (11.7)	19.8 (10.0)	MD −0.90 (−2.28 to 0.48)	0.199
ZDS (/100), mean (SD)	53.8 (24.4)	58.4 (25.9)	MD −0.26 (−3.66 to 3.14)	0.881

mRS, modified Rankin Scale; GCS, Glasgow Coma Scale; NIHSS, National Institutes of Health Stroke Scale; EQ-5D HUS, EuroQol 5-Dimensions Health Utility Score; EQ-VAS, EuroQol Visual Analogue Scale; Barthel Index, Barthel Index of Activities of Daily Living; TICS-M, Telephone Interview for Cognitive Status-Modified; ZDS, Zung Depression Scale.

**Fig. 2.**
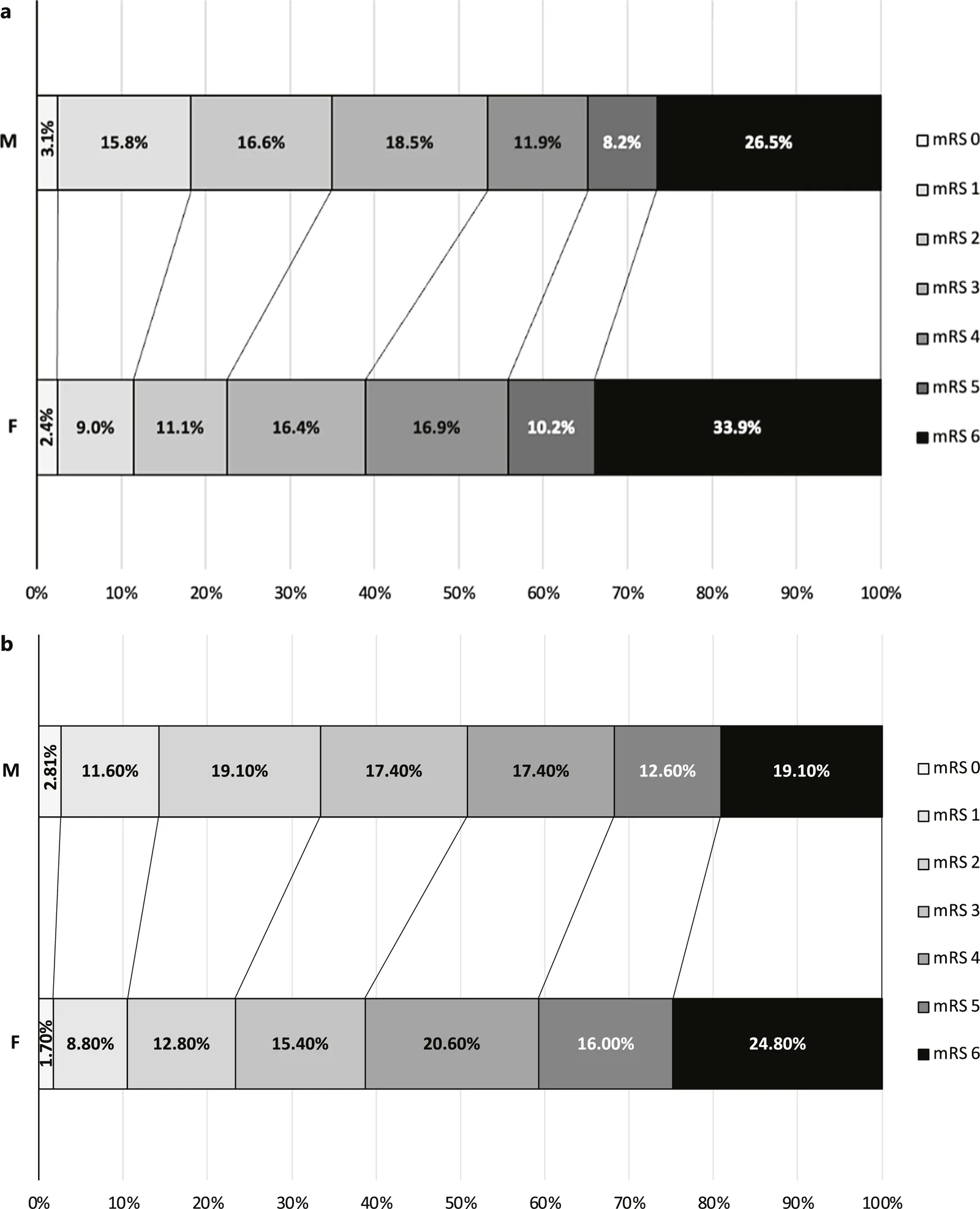
Shift analysis showing the distribution of 90-day (**a**), acOR 1.04 (95% CI 0.88–1.22, *p* = 0.602), and 1-year (**b**), acOR 1.05 (95% CI 0.87–1.23, *p* = 0.63), modified Rankin Scale (mRS) scores by sex. M, male; F, female.

**Fig. 3.**
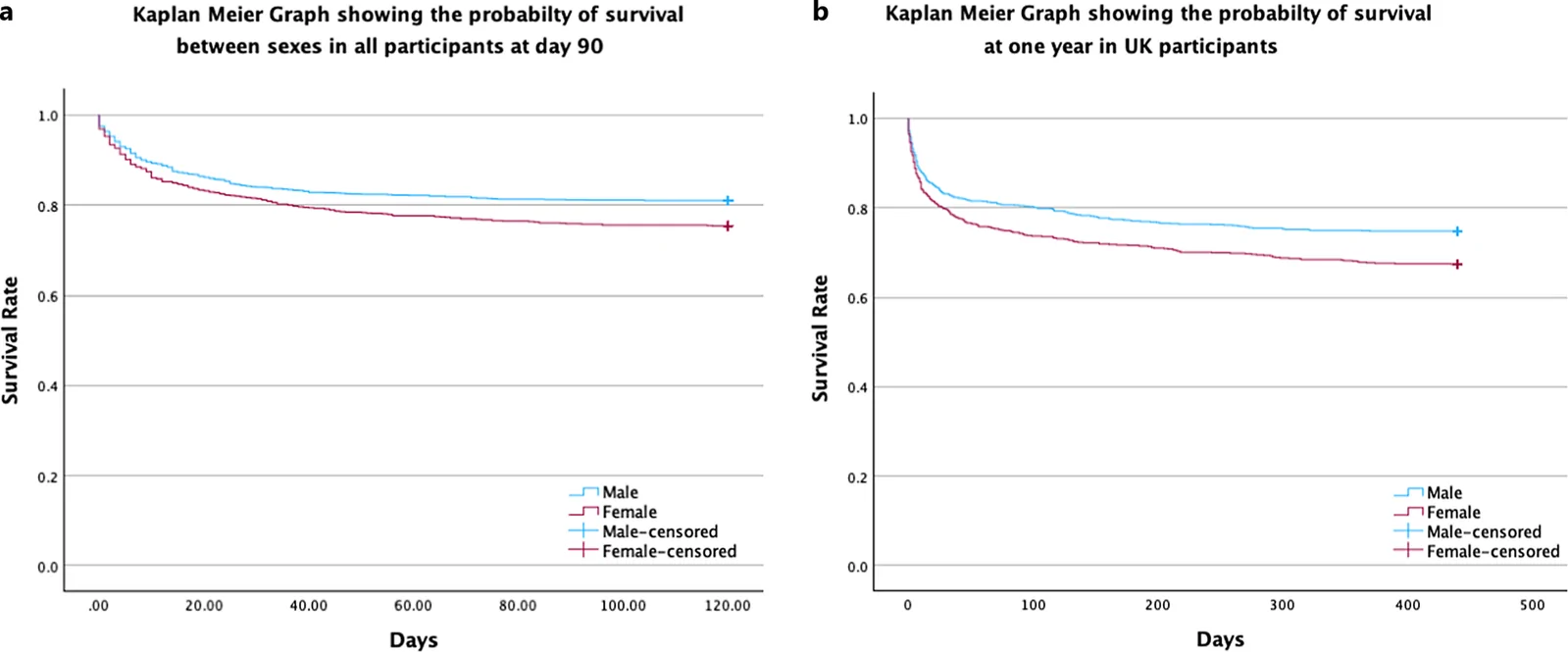
Kaplan-Meier survival curves comparing time to death between men and women at 90 (**a**), aHR 0.89 (95% CI 0.73–1.08, *p* = 0.23), and 1 year (**b**), aHR 0.87 (95% CI 0.72–1.04, *p* = 0.62).

Early neurological deterioration was similar between groups (Table 4). The proportion undergoing repeat CT at day 2 was similar across sexes (93.1% vs. 91.1%, *p* = 0.06) (Table 4). Interestingly, haematoma expansion was significantly less common in woman (25.3% vs. 28.2%, OR 0.77, 95% CI 0.62–0.95, *p* = 0.02) (Table 4) though other radiological markers, including perihaematomal oedema, midline shift, and hydrocephalus, were more frequent in women (Table 2).

The rate of serious adverse events up to day 90 was comparable (504/1,024 [49.2%] in women vs. 573/1,301 [44.0%] in men, *p* = 0.44) (Table 4). At the time of discharge, discharge destinations grouped as “home,” “institution,” and “in hospital” remained similar between the sexes. At follow-up, women were significantly more likely to be in an institution at day 90 (12.0% vs. 4.7%, OR 1.67, 95% CI 1.11–2.51, *p* = 0.01), although by day 365 this difference was no longer statistically significant (Table 4).

In the secondary analysis of outcomes at 365 days, a total of 1910 participants were eligible for analysis (UK participants only) (Fig. 1). At 365 days, the adjusted OR for the mRS distribution was 1.05 (95% CI 0.87–1.23, *p* = 0.63) (Table 4; Fig. 2 b). Mortality at 365 days was 32% vs. 25%; HR at 365 days 0.87 (95% CI 0.72–1.04, *p* = 0.62,1873 participants) (Table 4; Fig. 3 b).

Quality-of-life outcomes (in EQ-5D utility scores), independence with activities of daily living (Barthel Index), depression scores (ZDS), and cognition (TICS-M) were worse in women at 90 and 365 days, but after adjustment there were no significant differences (Table 4). There was no evidence of a sex-specific treatment effect with TXA. The interaction between sex and treatment allocation was nonsignificant for early death (*p* = 0.19), haematoma expansion (*p* = 0.89), and mRS at day 90 (*p* = 0.99), suggesting that the effect of TXA was consistent across both sexes (Fig. 4).

**Fig. 4.**
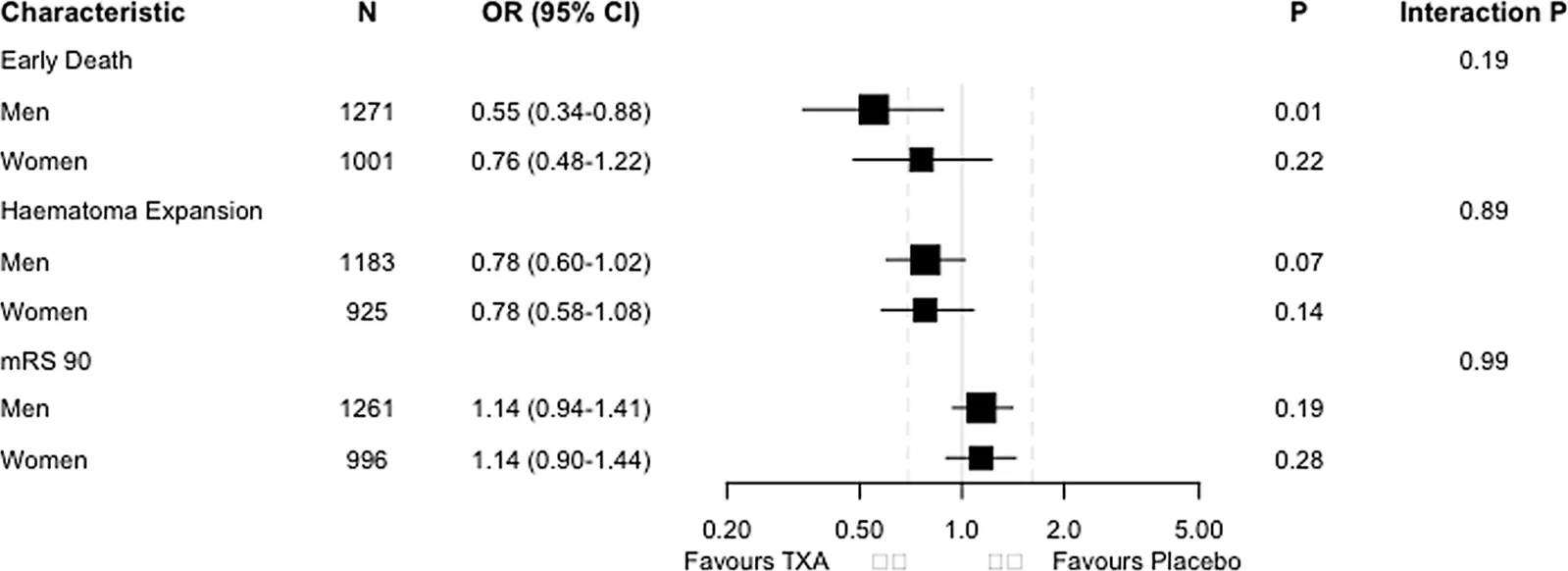
Subgroup analysis of the effect of tranexamic acid (TXA) versus placebo by sex for three key outcomes: early death, haematoma expansion, and 90-day functional outcome (mRS 0–6).

## Conclusion

In this post hoc analysis of the TICH-2 trial, women presented with several adverse prognostic features, including older age, worse pre-stroke function, more comorbidities, worse brain health on imaging with larger and lobar haematomas. After adjusting for these factors, sex was not associated with worse functional outcomes at 90 days or 1 year. Time from symptom onset to randomisation did not differ between sexes, suggesting that outcome variation relates more to baseline health and care decisions than to treatment delays. In processes of care, women were significantly more likely to have an early DNAR status in situ compared to men. Furthermore, the effect of TXA did not differ between sexes, consistent with sex-disaggregated findings from the CRASH trials on TXA use in traumatic brain injury [21].

Women with ICH are typically older and more likely to have lobar bleeds, likely due to cerebral small vessel disease and cerebral amyloid angiopathy. Men, by contrast, more commonly had deep haemorrhages, consistent with hypertensive vasculopathy. These differences in risk factor profiles and haemorrhage location are consistent with prior literature [10, 12, 22, 23]. However, unlike a recent meta-analysis showing unfavoured functional outcomes for women after ICH, after adjustment [10]. This likely reflects methodological differences. The meta-analysis pooled predominantly retrospective observational studies with variable outcome definitions and limited adjustment for confounding. Our findings are consistent with other prospective studies reporting no independent association between sex and functional outcome after accounting for baseline differences [12]. Previous studies, predominantly involving ischaemic or mixed stroke populations, have reported poorer health-related quality of life among women [24, 25]. These differences may be influenced by age, pre-stroke function, stroke severity, comorbidity, and socioeconomic or social factors [26]. In contrast, we found no independent sex difference in health-related quality of life after adjustment for measured baseline characteristics. Differences in stroke subtype, study populations, outcome assessment, and covariate adjustment may partly explain these apparently inconsistent findings. However, relevant factors including frailty, socioeconomic circumstances, and access to rehabilitation were not captured in TICH-2, and residual confounding cannot be excluded.

Women were more likely to receive early DNAR orders. These patterns persisted after adjusting for prognostic markers such as age and stroke severity, raising questions about how escalation decisions are made. While men more often received treatment in intensive care including intubation and ventilation, the difference was not significant, and this did not translate into better outcomes. These differences in care may reflect differing thresholds for intervention. Prior studies have linked early DNAR decisions with limited care escalation and increased mortality [27, 28]. Our findings highlight the need for further research to better understand how early resuscitation decisions influence subsequent management and longer-term outcomes after ICH.

This study has several strengths. It is the first study to report discrepancies in DNAR decisions in women. It draws on a large, robust international randomised controlled trial with consistent protocols and blinded outcome assessment. Access to patient-level data enabled robust adjustment for confounders and rare exploration of acute care decisions by sex.

Limitations include the secondary nature of this post hoc analysis and the potential for residual confounding despite adjustment for prespecified prognostic variables as unmeasured or incompletely captured factors may have influenced the observed associations. Accordingly, the absence of an independent association between sex and functional outcomes should be interpreted with appropriate caution. The study was not powered to detect sex-based outcome differences or sex-by-treatment interactions, and although no interaction between sex and TXA was observed, modest sex-specific treatment effects cannot be excluded. As participants were recruited into a randomised clinical trial, selection bias may also have occurred, with patients presenting late, lacking capacity to consent, or with severe comorbidities potentially underrepresented, limiting generalisability to routine clinical practice.

Key risk factors for older women such as frailty, menopausal status, pregnancy history, hormonal therapy, socioeconomic factors including education, occupation, income, and rehabilitation access were not collected and therefore cannot be excluded; there may also be residual cofounding due to this [2, 5, 29]. The largely Caucasian study population may also limit generalisability of the observed results.

The observed sex differences in baseline characteristics and care escalation, particularly older age, lobar haemorrhage, markers of poor brain health, and higher early DNAR use in women, have direct implications for clinical practice and research. Preventive strategies should reflect the different causes and risk factors of ICH seen in women and men, rather than relying on a single approach for all patients. Future trials and registries should routinely capture sex-relevant variables such as pre-stroke disability, frailty, and socioeconomic factors and explicitly examine how these interact with acute decision-making, including DNAR and ICU admission. Longitudinal analyses linking early care decisions to longer-term disability and discharge destinations are needed to determine whether observed differences in management contribute to outcomes.

## Acknowledgments

We thank all the sites’ principal investigators and participants that were involved in the TICH 2 trial.

## Statement of Ethics

This study was a secondary analysis of data from the Tranexamic Acid for Hyperacute Primary Intracerebral Haemorrhage (TICH-2) trial. The TICH-2 trial protocol was reviewed and approved by the East Midlands – Nottingham 2 Research Ethics Committee (REC reference: 12/EM/0369). The present study used pseudonymised participant-level data collected within the approved trial framework. Additional ethical approval was not required for this secondary analysis in accordance with applicable institutional and regulatory requirements. The study was conducted in accordance with the principles of the Declaration of Helsinki. Written informed consent for participation in the TICH-2 trial was obtained from all participants or, where participants lacked capacity, from an appropriate legal representative, relative/next of kin, or an independent physician acting in the participant’s best interests, in accordance with local regulations. Participants consented to the use of their anonymised data for future research and data sharing, including secondary analyses such as the present study.

## Conflict of Interest Statement

C.S.M., E.G., and M.D. have nothing to declare. K.K. supported with blinded analyses of scans for TICH-2. Z.K.L., L.W., and R.D. contributed to the TICH-2 trial. P.M.B. contributed to TICH-2, holds NIHR and BHF grants paid to the University of Nottingham and stock options in CoMind, receives lecture/consulting fees from Phagenesis, Roche, and DiaMedica, and is Industry Committee Co-Chair, WSC. N.S. holds research grants for TICH-2, DASH, and TICH-3 trials.

## Funding Sources

This post hoc analysis received no funding. The primary trial (TICH 2) was funded by National Institute of Health Research Health Technology Assessment Programme and Swiss Heart Foundation.

## Author Contributions

C.S.M.: conceptualisation, methodology, formal analysis, data interpretation, manuscript drafting, and revision. M.D., K.K., P.M.B., and N.S.: study conceptualisation, funding acquisition, supervision, data interpretation, and manuscript revision. E.G., Z.K.L., L.W., and R.D.: manuscript revision.

## Data Availability

The data that support the findings of this study are not publicly available due to ethical and governance restrictions. De-identified participant-level data may be made available on reasonable request to the corresponding author, subject to approval by the TICH-2 trial investigators and relevant institutional data governance processes.
